# Probiotic yogurt supplemented with nanopowdered eggshell: Shelf‐life stability, physicochemical, and sensory characteristics

**DOI:** 10.1002/fsn3.2152

**Published:** 2021-01-28

**Authors:** Dalia G. Kamel, Aly A. Othman, Dina M. Osman, Ahmed R. A. Hammam

**Affiliations:** ^1^ Dairy Science Department Faculty of Agriculture Assiut University Assiut Egypt; ^2^ Department of Physics Faculty of Science Assiut University Assiut Egypt; ^3^ Dairy and Food Science Department South Dakota State University Brookings SD USA

**Keywords:** functional properties, nanopowdered eggshell, probiotic yogurt, shelf‐life

## Abstract

The objectives of this study were to produce probiotic yogurt (5.0–7.0 log cfu/g) fortified with nanopowdered eggshell (NPES) at a rate of 0.02, 0.04, and 0.06 mg/ml, as well as, examine the effect of NPES on the physicochemical, microbial, sensory properties, and shelf‐life of probiotic yogurt. The NPES was prepared by milling preboiled dried eggshell using a mortar grinder. The size of the milled powder was measured to assure that the diameter of the powder is 27 ± 1.7 nm. Yogurt was manufactured by dividing the pasteurized milk into four aliquots portions. The first portion was utilized as control (T1), while the other three portions were supplemented with 0.02 (T2), 0.04 (T3), and 0.06 (T4) mg/ml NPES. All treatments were inoculated with 5.11 log cfu of *Lactobacillus delbruckii* ssp. *bulgaricus* (Lb) and *Streptococcus thermophilus* (St) combined and 5 log cfu of *Bifidobacterium bifidum* (Bb) per kg of milk at 40°C until the pH of 4.6 was reached. The acidity, sensory properties, Bb count, total bacterial count (TBC), yeast, and mold counts were examined. The results showed that the acidity was increasing during storage, however, increasing NPES resulted in low acid development (*p* < .05). The shelf‐life of control was ended after 8 d of storage at 4°C because molds were grown on the surface of the sample. The TBC significantly decreased (*p* < .05) as the concentration of NPES increased. Bb count in probiotic yogurt was also decreasing during storage. Yeast and molds were detected in control after 8 d; however, NPES did not result in molds even after 16 d of storage but yeast was exhibited. The NPES improved the sensory evaluation of probiotic yogurt slightly and increased the shelf‐life of probiotic yogurt as compared to control.

## Graphical abstract

The nanopowdered eggshell can be used to improve the sensory evaluation of probiotic yogurt slightly and increased the shelf‐life of probiotic yogurt.

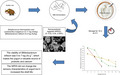

## 1 INTRODUCTION

Eggshell (ES) is a waste product for the food industry that could increase environmental pollution. The ES is an alternative, cheap, and bioavailable source of dietary calcium. ES has about 39% of elemental calcium, which has a higher bioavailability compared to calcium carbonate (Schaafsma & Beelen, 1999). Calcium in ES strengthens the bones and prevents osteoporosis, especially in postmenopausal women (Makai & Chudacek, 1991; Schaafsma & Pakan, 1999). There is also other nutrients in ES, including magnesium, phosphorus, glycoproteins, and proteoglycans (Hincke, 1995; Hincke et al., 1999).

Nanotechnology is a novel process that can be used to improve the physicochemical and biological properties of dairy products. Nano sizing particles play a significant role in increasing the bioavailability of the microcomponent, such as calcium (Hilty et al., 2011; Park et al., 2008; Seo et al., 2009). As a result, nanopowdered eggshell (NPES) can be used as nanoparticles in the manufacture of probiotic yogurt. NPES has a potential application in manufacture of yogurt using probiotics. It has been found that NPES did not show any negative effects on the characteristics of yogurt (Al Mijan et al., 2014).

In 2003, Codex mentioned that probiotic dairy products should contain at least 6–7 log cfu/g of at the time of consumption in quantity higher than 100 g per d to have at least 9 log cfu per d (FAO/WHO, 2010). Probiotic bacteria, including *Bifidobacterium bifidus,* has several benefits, such as improving the gastrointestinal tract. This can also reduce acute diarrhea and *E. coli* infections (Caballero et al., 2015). Additionally, it has been presented that the NPES can be utilized to improve the growth of probiotic bacteria in yogurt during storage (Al Mijan et al., 2014). The objectives of this work were to produce probiotic yogurt (5.0–7.0 log cfu/g) fortified with NPES at a rate of 0.02, 0.04, and 0.06 mg/ml, as well as, study the effect of NPES on the physicochemical, microbial, sensory properties, and shelf‐life of probiotic yogurt.

## 2 MATERIAL AND METHODS

### 2.1 Manufacture of probiotic yogurt

Fresh buffalo's milk was obtained from the Animal Farm (Faculty of Agriculture, Assiut University, Assiut, Egypt), heated to 90°C for 5 min, and cooled to 40°C. The milk was inoculated with 5.11 log cfu of *Lactobacillus delbruckii* ssp. *bulgaricus* (Lb) and *Streptococcus thermophilus* (St) (Dairy Science Department, Faculty of Agriculture, Assiut University, Egypt) combined, and 5 log cfu of *Bifidobacterium bifidum* (Bb) per kg of milk (Cairo MIRCEN, Faculty of Agriculture, Ain Shams University, Egypt). The milk was divided into 4 aliquots portions. The first portion was utilized as control (T1; with no NPES) while 0.02, 0.04, and 0.06 mg/ml of NPES were added to the second (T2), third (T3), and fourth (T4) portions, respectively. All treatments were inoculated at 40°C until a pH of 4.6 was reached and this process took approximately 4 hr. Subsequently, the yogurt was cooled and stored at 4°C for 16 d. This experiment was repeated 3 times using 3 different batches of raw milk.

### 2.2 Preparation of nanopowdered eggshell (NPES)

ES was collected from domestic sectors in Assiut governorate, Egypt. ES was washed thoroughly with warm water and dried at room temperature for 2 d. After drying, it was kept in boiling water for 2 hr to remove the interior membranes as well as undesirable substances. ES was then dried in the oven at 60°C for 6 hr. Further, the dried ES was milled using mortar grinding Fritsch Pulversitte 2, for one h. The X‐ray diffraction (XRD) of the milled powder was recorded using PW1700 X‐ray diffractometer in the 2θ range from 20°C to 50°C. The mean crystallite size D of the obtained NPES was calculated by using the Scherrer Equation (1):

(1) D=0.89λ/βSinθ

where *λ* is the CuK_α_ x‐rays (1.54056 A°), β is the full width at half maximum of the diffraction peak, Ɵ is the different angle. The mean crystallite size D was found to be 27 ± 1.7 nm.

### 2.3 Chemical and microbiological analyses

Titratable acidity was determined by calculating the lactic acid content in the yogurt (Akın et al., 2007; Sadler & Murphy, 2010). Total bacterial count (TBC), *Bifidobacterium bifidum* (Bb) count, yeast, and mold counts were determined as described by Hamdy and others (Hamdy et al., 2021). The chemical and microbiological analyses were performed at 0, 4, 8, 12, and 16 d.

### 2.4 Sensory evaluation

Sensory evaluation of probiotic yogurt was also determined as described by Hamdy and others with some modifications (Hamdy et al., 2021). Samples were evaluated for color and appearance (15 points), flavor (45 points), acidity (10 points), body and texture (30 points) to have 100 points as a total. The sensory characteristics were determined at 0 and 16 d.

### 2.5 Statistical analysis

Data were statistically analyzed using R software (R x64‐3.3.3, 9,205 NW 101st St, Miami, Florida, United States) by ANOVA using a GLM for each variable to study the effect of NPES and time or their interaction on the characteristics of probiotic yogurt. Mean separation was done using the least significant difference (LSD) comparison test when significant differences were detected at *p* < .05.

## 3 RESULTS AND DISCUSSION

### 3.1 Particle size analysis

**FIGURE 1:**
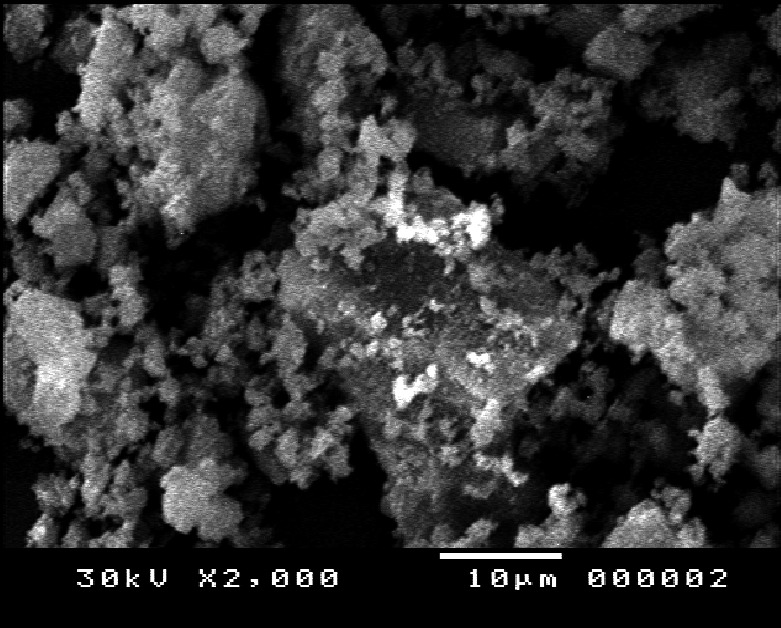
Scanning electron microscopy (*SEM*) micrographs of nanopowdered eggshell powder (NPES)

**FIGURE 2:**
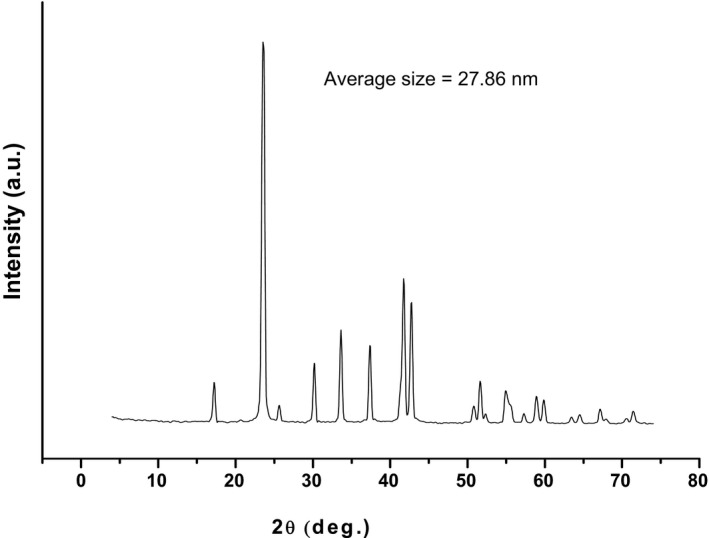
Particle size distributions of nanopowdered eggshell powder (NPES) in the x‐ray diffraction (XRD)

The morphology of NPES was observed by scanning electron microscopy (*SEM*; model JEOL JSM‐5400 LV), as shown in Figure 1. The *SEM* demonstrated that the average particle size for NPES was about 18 to 20 µ and there are on the nanoparticles (20 to 40 nm). Figure 1 illustrates that *SEM* shows that the NPES consists of nanosized crystals of particles. Figure 1 presented that the nanoparticles agglomerate to form the large particles. This agglomeration accompanies the sample preparation for recording the *SEM* image. Also, Figure 2 shows the XRD pattern of the NPES. Figure 2 indicates the nanocrystalline of the NPES. The average crystal size (D) was calculated by using Scherrer Equation (2), thus D = 27±1.7 nm:

(2) D=kλ/βCosθ

### 3.2 Titratable acidity (% lactic acid)

**FIGURE 3:**
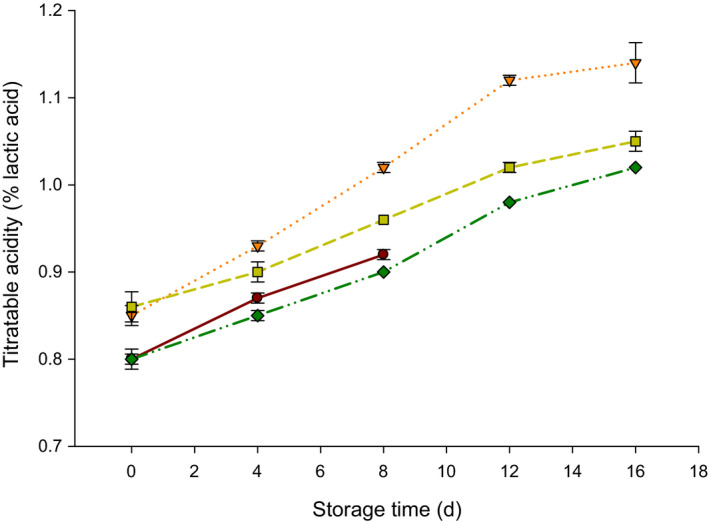
Acidity (%) of probiotic yogurt supplemented with 0.0 (●), 0.02 (▼), 0.04 (■), and 0.06 (♦) mg/ml nanopowdered eggshell powder (NPES) stored at 4°C for 16 d

The changes in the acidity of probiotic yogurt supplemented with different concentrations of NPES during 16 d of storage at 4°C are presented in Figure 3. All probiotic yogurt samples gradually increased in acidity during the storage period. The acidity values in 0.02, 0.04 and 0.06 mg/ml NPES yogurt ranged from 0.85 to 1.14, 0.86 to 1.05, and 0.80 to 1.02%, respectively through the 16 d of storage, whereas it ranged from 0.80% to 0.92% after 8 d of storage in the control probiotic yogurt. However, increasing the concentrations of NPES in probiotic yogurts resulted in a decrease in the acidity value. From Figure 3, it shows that the average of acidity in T4 (0.06 mg/ml NPES) was lower than the acidity in T2 and T3. This can be due to the higher buffering capacity of calcium in NPES.

The obtained results are in agreement with the results obtained by Pirkul et al. (1997) who found that higher calcium in yogurts had higher pH values than the control. It turns out that the buffering activity of the calcium present in the ES probably contributed to the higher values of acidity. Also, similar results were obtained by other researchers when they added NPES to probiotic yogurt (Al Mijan et al., 2014; El‐Shibiny et al., 2018). El‐Shibiny et al. (2018) found that the addition of NPES at >0.1% decreased acid development significantly (*p* < .05). The general trend of acidity in probiotic yogurt during storage was also similar to other studies (Kim et al., 1992; Vinderola et al., 2000).

### 3.3 Total bacterial count (TBC)

**FIGURE 4:**
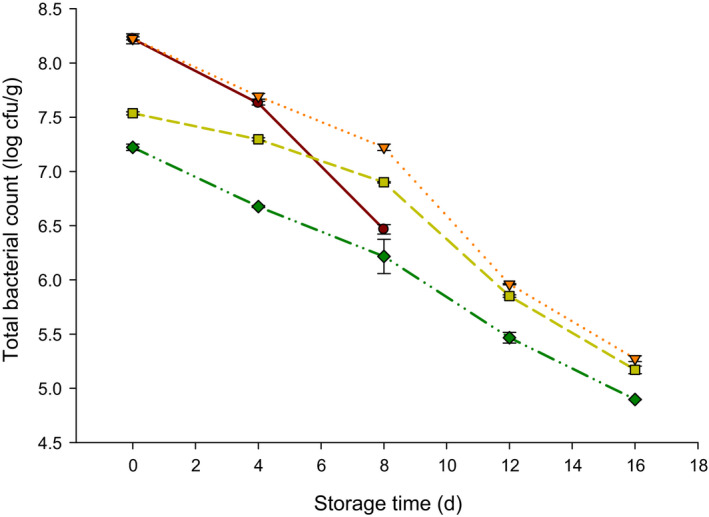
Total bacterial count (TBC; log cfu/g) of probiotic yogurt supplemented with 0.0 (●), 0.02 (▼), 0.04 (■), and 0.06 (♦) mg/ml nanopowdered eggshell powder (NPES) stored at 4°C for 16 d

The TBC of probiotic yogurt made with NPES is shown in Figure 4. The shelf‐life of control was finished at 8 d since the molds were noticeable on the surface of the yogurt. As a result, the last reading of TBC in control samples was recorded at 8 d. However, the TBC was decreased in all yogurt samples during storage at 4°C. The TBC in yogurt with no added NPES decreased from approximately 8.22 to 6.46 log cfu/g after 8 d. The TBC was lower at 0 d in yogurt with NPES as compared to control. The TBC at 0 d was found 8.22, 7.53, and 7.22 log cfu/g when 0.02, 0.04, and 0.06 mg/ml of NPES were added to the probiotic yogurt, respectively, and these values decreased to 5.27, 5.17, and 4.89 log cfu/g, respectively, after 16 d of storage. These results showed that the 0.06 mg/ml NPES resulted in lower TBC. The NPES can act as an antibacterial agent. These results are similar to another study that reported calcium carbonate nanoparticles can be used as an antimicrobial agent (Ataee et al., 2011). Al Mijan et al. (2014) have found that ES powder enriched with a high level of calcium carbonate has antimicrobial properties and indicated NPES supplemented yogurt has an extended shelf‐life.

### 3.4 Bifidobacterium bifidum (Bb)

**FIGURE 5:**
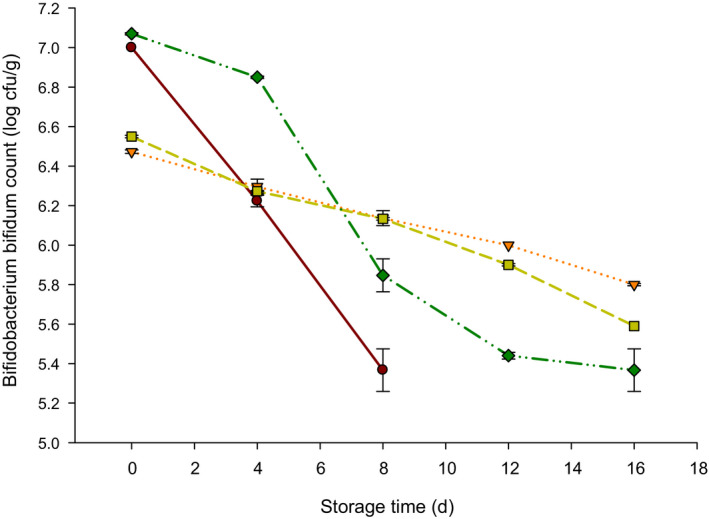
*Bifidobacterium bifidum* count (Bb; log cfu/g) of probiotic yogurt supplemented with 0.0 (●), 0.02 (▼), 0.04 (■), and 0.06 (♦) mg/ml nanopowdered eggshell powder (NPES) stored at 4°C for 16

The Bb count in probiotic yogurt is presented in Figure 5. The Bb in control decreased from 7 to 5.36 log cfu/g after 8 d of storage (end of storage for control due to growth of molds). Also, this number decreased from 6.47 to 5.8 log cfu/g in T2 (0.02 mg/ml of NPES), 6.55 to 5.59 log cfu/g in T3 (0.04 mg/ml of NPES), and 7.07 to 5.36 log cfu/g in T4 (0.06 mg/ml of NPES) after 16 d of storage at 4°C. All NPES treatments maintained 5 to 7 log cfu/g Bb in probiotic yogurt.

Decreasing the Bb count in probiotic yogurt supplemented with NPES can be due to the antimicrobial effect of calcium obtained from ES (Ataee et al., 2011). However, another study reported that lactic acid bacteria count in yogurt fortified with NPES was elevated during storage (Al Mijan et al., 2014). This trend can be due to the differences in the acidity level.

### 3.5 Yeast and mold count

**FIGURE 6:**
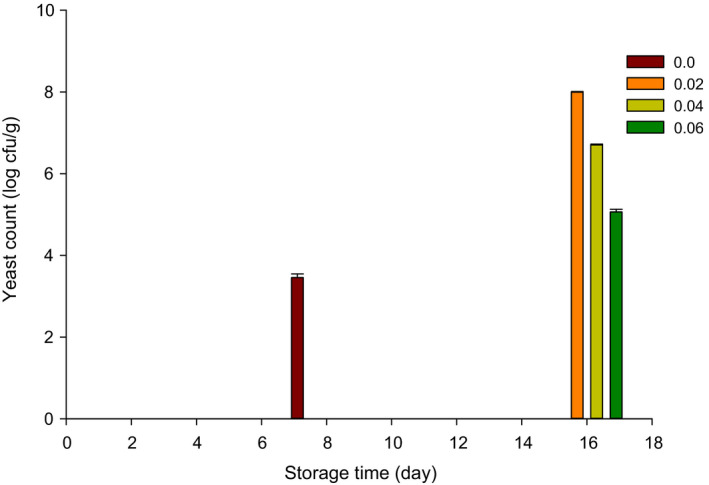
Yeast count (log cfu/g) of probiotic yogurt supplemented with 0.0, 0.02, 0.04, and 0.06 mg/ml nanopowdered eggshell powder (NPES) stored at 4°C for 16 d

The yeast count of probiotic yogurt is presented in Figure 6. The yeast counts were not detected in control up to 8 d of storage, while it was detected in yogurt supplemented with 0.02, 0.04, and 0.06 mg/ml of NPES after 16 d of storage. The molds were detected in control after 8 d while yogurt supplemented with NPES did not exhibit any molds. The yeast count was 3.45 log cfu/g in control after 8 d. However, the value of yeast was 7.99, 6.70, and 5.06 log cfu/g after adding of 0.02, 0.04, and 0.06 mg/ml of NPES, respectively, after 16 d of storage at 4°C. It looks like the NPES has decreased the growth of yeast, which increases the shelf‐life or storage time of probiotic yogurt. The yeast count significantly decreased with elevating NPES levels. That was due to the antimicrobial effect of calcium (Ataee et al., 2011).

### 3.6 Sensory evaluation

The sensory evaluation of probiotic yogurt is presented in Table 1. The NPES did not significantly change (*p* > .05) the sensory characteristics of yogurt during the storage time. On the first day, the acidity score decreased with increasing concentration of NPES. Other studies have reported similar results that different concentrations of calcium in the yogurt resulted in a higher pH value (Al Mijan et al., 2014) due to the buffering activity of the calcium presented in the NPES obtained from nano sizing (Pirkul et al., 1997). Al Mijan et al. (2014) reported that the addition of NPES has a substantial effect in increasing the shelf‐life of yogurt and this is compatible with our results. We noticed that the flavor is similar in all treatments, and the flavor in NPES yogurt was acceptable to the panelist. These results were in agreement with Al Mijan et al. (2014). There was a slight increase in the scores of texture and appearance at 0 d as compared to control. The control was not judged at 16 d due to the short shelf‐life of that treatment that ended after 8 d. The overall scores tend to increase after 16 d of storage and this might be due to the high amount of calcium in NPES, which resulted in a firmer texture. Singh and Muthukumarappan (2008) reported that the fruit yogurts enriched with calcium were not different from the control in terms of appearance, flavor, texture, and overall acceptability. Additionally, these results are in agreement with El‐Shibiny et al. (2018) who reported that the addition of up to 0.3% NPES made from cow and buffalo was acceptable and had higher quality.

**TABLE 1:** Sensory evaluation of probiotic yogurt made with nanopowdered eggshell powder (NPES) at a rate of 0.0, 0.02, 0.04, and 0.06 mg/ml

Treatment^a^	Time (d)	Flavor	Texture	Appearance	Acidity	Overall score
0.00	0	43	27	11.00	10.00	91.00
0.02		43	28	12.00	9.00	92.00
0.04		43	28	13.00	8.00	92.00
0.06		43	28	14.00	7.00	92.00
0.00	16	ND	ND	ND	ND	ND
0.02		42	28	14.00	8.00	92.00
0.04		43	29	14.00	7.00	93.00
0.06		44	30	14.00	6.00	94.00

ND, not determined.

^a^ Treatment = nopowdered eggshell powder (NPES): 0.0 = Control; T1 = 0.02 mg/ml; T2 = 0.04 mg/ml; T4 = 0.06 mg/ml

## 4 CONCLUSION

NPES can be used to manufacture probiotic yogurt to improve the physicochemical, microbial properties, and shelf‐life stability without any effects on the sensory properties. Therefore we conclude that the addition of 0.06 mg/ml NPES could be applicable to manufacture probiotic yogurt with an acceptable composition and quality as compared to control. The addition of NPES increased the shelf‐life of probiotic yogurt as compared to control with a range of 5 to 7 log cfu/g of probiotic yogurt.
